# Study on Physicochemical Properties of Biocomposite Films with Spent Coffee Grounds as a Filler and Their Influence on Physiological State of Growing Plants

**DOI:** 10.3390/ijms24097864

**Published:** 2023-04-26

**Authors:** Magdalena Zdanowicz, Marta Rokosa, Magdalena Pieczykolan, Adrian Krzysztof Antosik, Justyna Chudecka, Małgorzata Mikiciuk

**Affiliations:** 1 Center of Bioimmobilisation and Innovative Packaging Materials, Faculty of Food Sciences and Fisheries, West Pomeranian University of Technology in Szczecin, Janickiego St. 35, 71-270 Szczecin, Poland; 2 Laboratory of Plant Physiology and Entomology, Department of Bioengineering, Faculty of Environmental Management and Agriculture, West Pomeranian University of Technology in Szczecin, Słowackiego St. 17, 70-953 Szczecin, Poland; marta.rokosa@gmail.com (M.R.) Malgorzata.Mikiciuk@zut.edu.pl (M.M.); 3 Department of Chemical Organic Technology and Polymeric Materials, Faculty of Chemical Technology and Engineering, West Pomeranian University of Technology in Szczecin, Piastow Ave. 42, 71-065 Szczecin, Poland; 4 Department of Environmental Management, Faculty of Environmental Management and Agriculture, West Pomeranian University of Technology in Szczecin, Słowackiego St. 17, 71-434 Szczecin, Poland

**Keywords:** biocomposites, coffee grounds, deep eutectic solvents, fertilization, thermoplastic starch, urea

## Abstract

The aim of the study was to plasticize corn starch with two selected urea (U)-rich plasticizers: choline chloride (CC):U or betaine (B):U eutectic mixtures at a molar ratio of 1:5 with a presence of spent coffee grounds as a filler. The biomaterials were prepared via a solventless one-step extrusion method and then extrudates were thermoformed using compression molding into sheets. The materials were characterized using mechanical and sorption tests, TGA, DMTA and FTIR. Additionally, a study on the biodegradation and remaining nitrogen content in soil was conducted. For the first time, an influence on physiological state of growing plants of the materials presence in soil was investigated. The addition of the coffee filler slightly increased the mechanical properties and decreased the swelling degree of the materials. The DMTA results indicated that biocomposites were easily thermoformable and the high filler addition (20 pph per polymer matrix) did not affect the processability. According to the biodegradation test results, the materials disappeared in soil within ca. 70 days. The results from this study on the physiological state of growing plants revealed that the materials, especially plasticized with CCU, did not exhibit any toxic effect on the yellow dwarf bean. The percentage of total nitrogen in the soil substrate in comparison with the control increased indicating an effective release of nitrogen from the TPS materials into the substrate.

## 1. Introduction

Starch as one of the most abundant biopolymers is widely used in many areas of industry. It can be applied in areas such as food technology, pharmacy, paper making and packaging [1]. Additionally, it can be processed as conventional oil-based plastics (i.e., via extrusion and thermocompression) such as polyolefins with the addition of plasticizers. The native form of the polysaccharide due to its temperature of degradation near the glass temperature (T_g_) is not processable; this is why polar small molecule plasticizers are added. They decrease the T_g_ of starch and lead to the swelling of granules, thus leading to amorphization of starch under high temperature, high pressure and shear forces enabling its processing to obtain transparent flexible thermoplastic starch that is fully biodegradable. The most common plasticizer to obtain TPS is glycerol but other polyols (e.g., sorbitol), amines, amides or sugars can also be used [2,3]. Lately, ionic liquids (ILs) and deep eutectic solvents (DESs) have been studied as starch modifiers [4,5,6,7]. DESs have several advantages over ILs: they are easy to prepare (only via heating of components at certain molar ratios) from cheap, available and non-toxic components. Many DESs that are effective plasticizers for starch are based on urea (U) [8,9,10,11]. Thus, TPS enriched with U can be used in the agriculture or horticulture industry as, for example, a new generation of fully biodegradable, natural-based fertilizers or agricultural bioplastics (e.g., flowerpots, mulches, seed tapes). In this work, mixtures of ammonium salts, namely, choline chloride (CC) or betaine (B) with urea at a 1:5 molar ratio, were used as plasticizing systems. The systems were selected based on our previous study [10], where results showed that a higher content of urea in DES can modify starch effectively.

The TPS plasticized with U-based DES [8,9,10,11] and other mixtures with U [12,13,14] exhibited low tensile strength, but high elongation at break and high swelling degree. One of the methods to affect mechanical and barrier properties of TPS is an introduction of fillers into the polysaccharide matrix. In this work, we chose to use spent coffee grounds (cf) to obtain natural-based biocomposites with some fertilizing functions. Coffee, as one of the most popular beverages around the world, generates highly valuable waste in the form of grounds that have a potential to be converted into high-value different bio-products of cf (even after brewing) containing high amounts of polysaccharides, sugars, oils, antioxidants, phenolic compounds (e.g., chlorogenic acid, gallic acid) [15,16,17] and other high value compounds (e.g., nutrients, such as calcium (1.5 g/kg), magnesium (19.0 g/kg), potassium (2.9 g/kg), sodium (4.5 g/kg), iron (0.05 g/kg), zinc (0.009 g/kg), manganese (0.03 g/kg), and copper (0.02 g/kg)) [18]. It is worth highlighting that cf contain ca. 1.8–2.8% nitrogen [16,19] from, e.g. proteins and caffeine. Thus, they are popular fertilizing additives even in the home cultivation of plants. Besides the application in agriculture [20], spent coffee grounds can be a source of energy, as well as used for oil, biodiesel and bioethanol production or be applied as adsorptive filters [16]. Moreover, they can be used as precursors for biopolymer production [21]. Coffee grounds have been added also as a filler into polymeric matrix both to bio-based and oil-based materials [21,22]. Nevertheless, there are only a few studies related to TPS with cf. However, these works present materials plasticized with glycerol [23,24] and most of them are related to TPS obtained via the casting method [23]. In this work, for the first time, TPS materials plasticized with DES enriched with urea with the presence of spent coffee grounds as a filler are presented. It is worth mentioning that there are only a few works presenting studies on extruded TPS/DES/fillers [25,26,27], so the work brings some development in this field.

In the presented work, thermoplastic starch films plasticized with two selected DESs, namely, choline chloride:urea (CCU) and betaine:urea (BU) at a molar ratio of 1:5, were obtained via twin-screw extrusion. Selected physicochemical properties of the biocomposites were investigated, i.e., mechanical and viscoelastic properties (using dynamic mechanical thermal analysis—DMTA) and thermal stability (thermogravimetry). The TPS/U-rich DES/cf systems have not been presented so far, so the mentioned techniques were used to investigate an influence of a high filler content (20 pph per polysaccharide) on the mechanical properties and processability of the biocomposites. Moreover, behavior in moisture and water as well as biodegradation degree in soil were determined. Due to the high content of U in the biomaterials, they are intended for plant cultivation. Thus, for the first time, the influence of the materials on the physiological state of growing plants (*Phaseolus vulgaris* L. was used as the model plant) was investigated, including the investigation of gas exchange parameters, chlorophyl “a” fluorescence parameters, assimilative pigments and proline concentration, to examine if the new materials exhibit any toxic effect on the plants. Additionally, nitrogen content in soil after cultivation was measured.

## 2. Results and Discussion

**Scheme 1:**
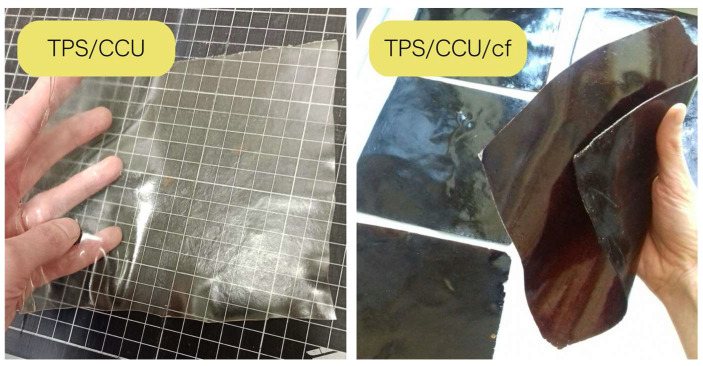
Thermoplastic corn starch plasticized with CCU without filler (**left**) and with addition of spent coffee grounds (cf) (**right**).

Scheme of film preparation according to two methods of premixture preparation is described and graphically illustrated in the Materials and Methods section. The TPS films without filler after thermocompression are highly transparent (Scheme 1), smooth and flexible; however, after a few days of storage for the films with the BU, a white layer is observed. This phenomenon is caused by urea recrystallization [28,29] in the polysaccharide matrix due to its excess in the plasticizing system.

### 2.1. Mechanical Tests Results

**Figure 1:**
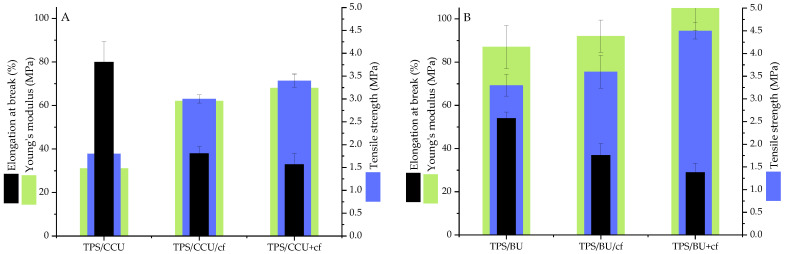
Mechanical properties of TPS plasticized with: (**A**)—CCU and (**B**)—BU.

Figure 1 shows the mechanical test results for the TPS films plasticized with two types of plasticizing mixtures (60 pph per dry starch) as well as the TPS composites with the addition of spent coffee grounds as fillers (20 pph per dry starch).

Comparing the plasticizers, the TPS/BU films exhibited a higher tensile strength (TS); however, the CCU had a higher elongation at break (Figure 1). These differences were caused by a better plasticizing efficiency of CCU without its recrystallization. Similar results were obtained in our previous study [10]. Both types of TPS filler presence reinforced the films leading to a slight increase in Young’s modulus, TS and decrease in EB. Notably, a high filler content (20 pph) did not lead to brittleness of the samples, what we can often observe for TPS with conventional plasticizers [30]. This phenomenon is caused by the presence of the eutectic system in the modified matrix [25,26]. Slightly better mechanical properties were obtained for samples where filler was heated in DES and then the DES/cf was added into starch (TS 3.4 MPa for TPS/CCU+cf and 4.5 MPa for TPS/BU+cf). These differences can be caused by the partial extraction and dissolution of compounds from the cf that migrated into DES and then into the polysaccharide matrix [31]. These organic compounds (i.e., polyphenols) can act as compatibilizers and increase an adhesion between filler particles and hydroxyl groups from the starch chain, i.e., in our previous study [26], the improvement in mechanical properties was obtained even if the filler was added as dissolved form in the DES. In Callazo-Bigliardi et al.’s work, the addition of extracts from coffee husks increased the mechanical properties. However, the TPS was obtained via a melt-blending process with a lower amount of glycerol as plasticizers, so the obtained materials with a high content of extract were quite brittle [32]. In the work of [26], the influence of the different cf content on the physicochemical properties of TPS/glycerol was studied and the improvement in mechanical properties was obtained for up to 10 pph of the filler, and the higher content of the parameters decreased.

### 2.2. Dynamical Mechanical Thermal Analysis (DMTA)

**Figure 2:**
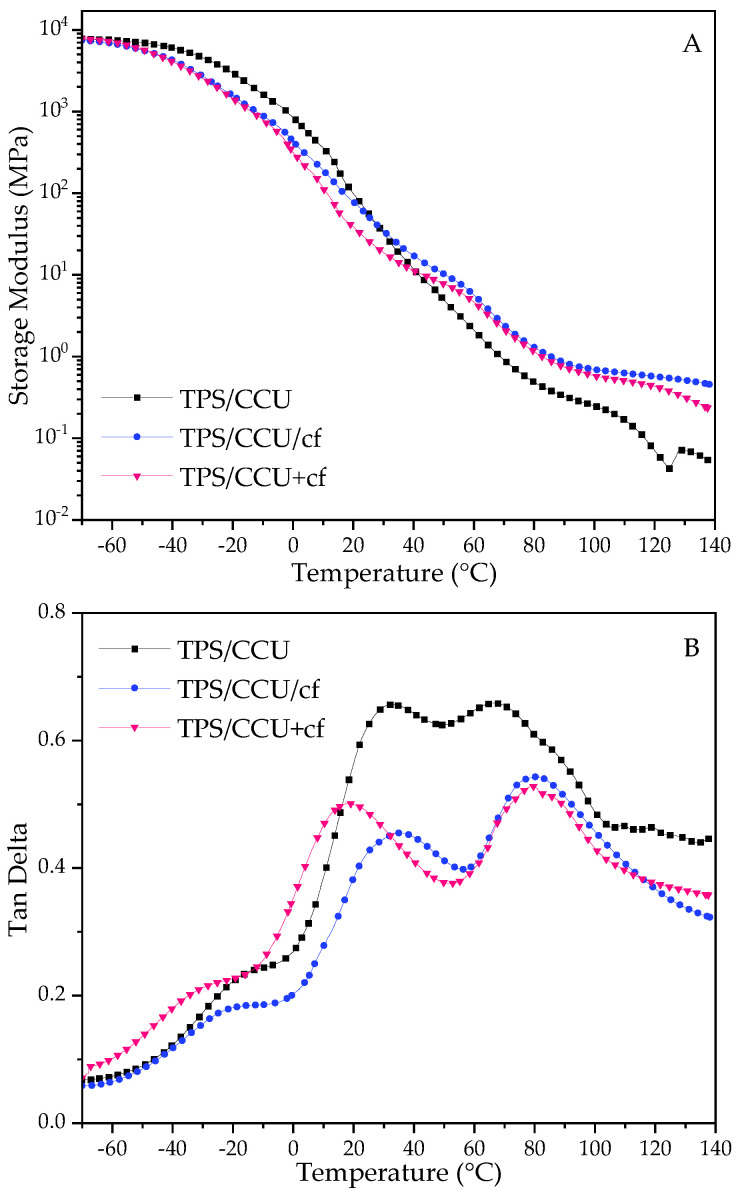

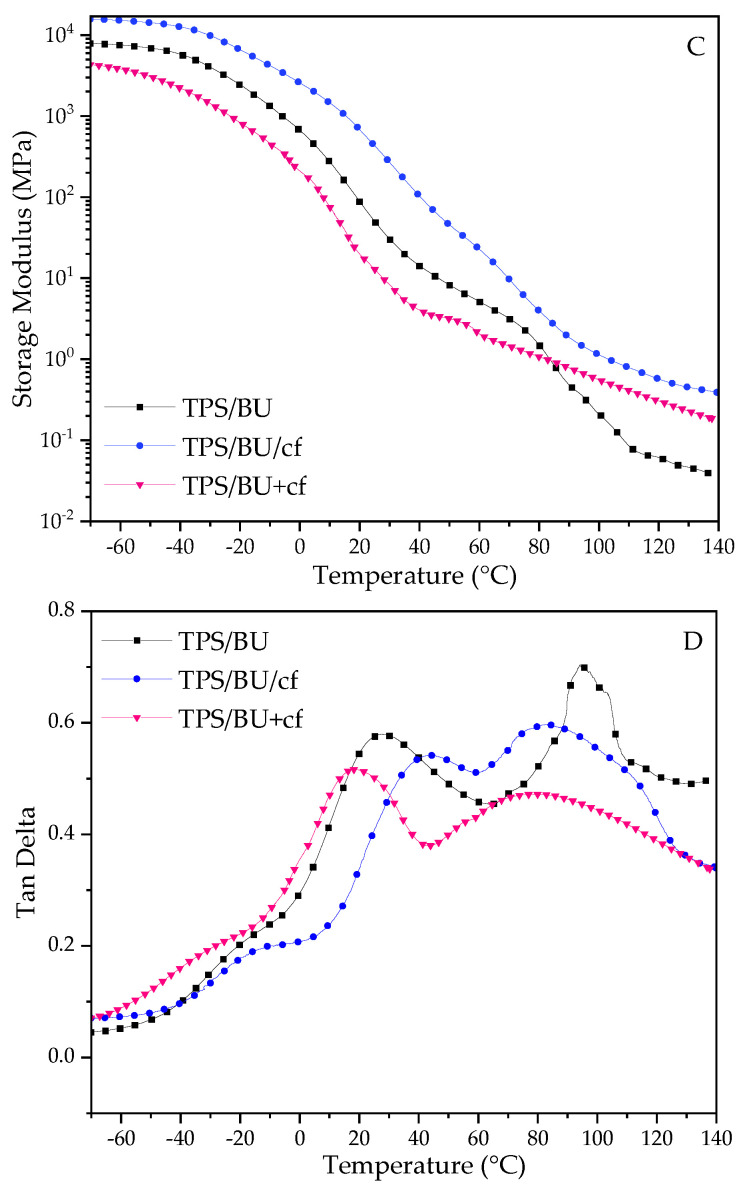
DMTA results for TPS films. Storage modulus for TPS with CCU—(**A**) and for TPS with BU—(**C**) and tanδ for TPS with CCU—(**B**) and for TPS with BU—(**D**).

For the DMTA results, the storage modulus (E′) and tan delta curves are depicted in Figure 2.

The storage moduli (E′) for the films rapidly decreased with the increase in temperature, and the values were low at elevated temperatures, indicating their ability for further thermoprocessing [25,26,27]. Comparing the two types of plasticizers, the drop in the E′ parameter is more prominent for CCU. It can be correlated with the mechanical test results and assigned to a better plasticizing effect of this DES. There is a difference in TPS films and their composite analogues. For TPS with CCU, E′ is lower than TPS with the filler above room temperature and above ca. 80 °C for BU. In the case of the films with CCU, there is no great difference between the composites. However, for TPS with BU, there is noticeable difference between TPS/BU/cf and TPS/BU+cf. It can indicate that DES/cf premixtures form stronger bonds between DES and coffee grounds by partial extraction compounds from cf. Tan delta curves revealed three relaxation peaks, the first one at a temperature below 0 °C is a β-relaxation assigned to the movement of small molecules such as water and external plasticizer, and for the second one, α-relaxation with high intensity came from the movement of plasticized polysaccharide chains, and the third peak is a α′-relaxation peak assigned to moisture evaporation from the materials [33]; these peaks did not appear for the dried samples. The intensity of the α-relaxation peak is lower for composites suggesting a restriction of the polymer chains movement with the filler presence. The temperature values of α-peak for both types of plasticizers are as follows: TPS/DES+cf < TPS/DES < TPS/DES/cf. Lower temperature for TPS/DES+cf may be related to cf modification with DES during the preheating and extraction of more mobile molecules with lower molecular weight from the cf into DES. The highest temperature for TPS/DES/cf is related to reinforcing the activity of solid filler [34]. The sharp peak at ca. 95 °C for TPS/BU+cf (Figure 2D) is assigned to some transition (melting) of the plasticizer component’s crystals.

### 2.3. Thermal Stability of TPS Films (TGA)

**Figure 3:**
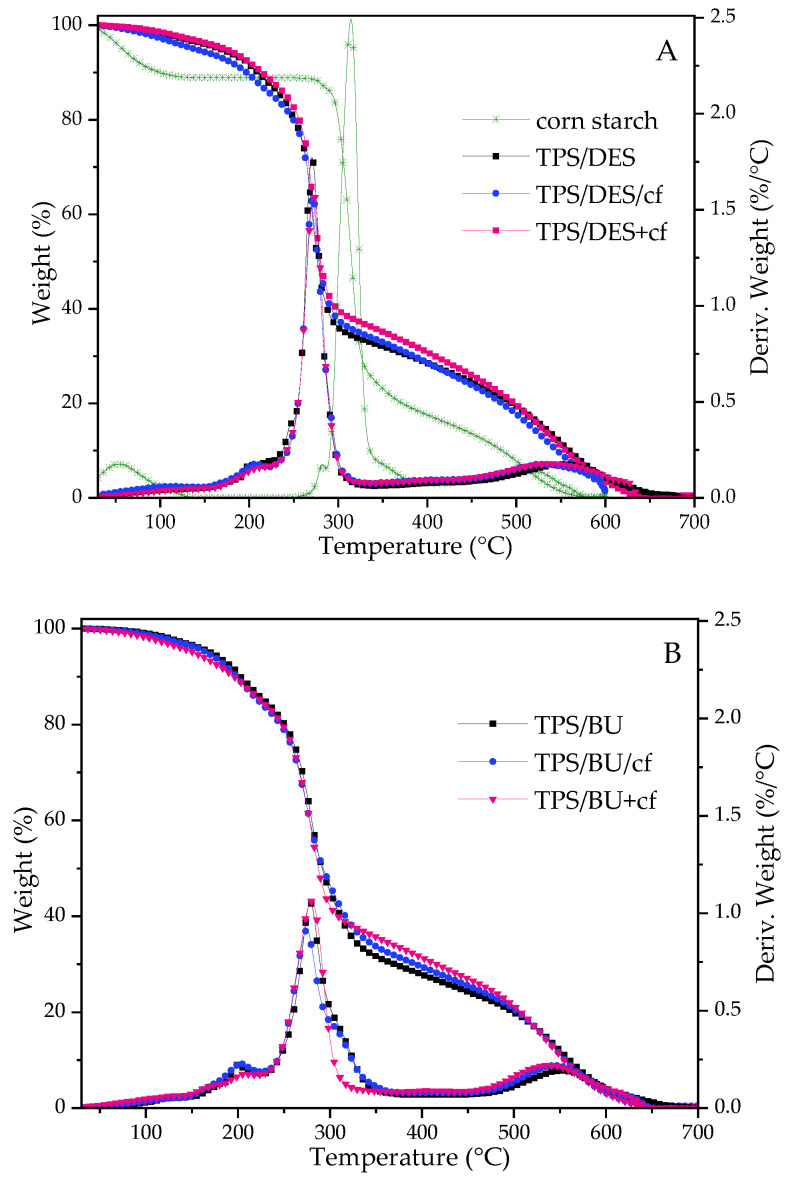
TGA and DTG curves for native corn starch, TPS/DES and TPS/DES/filler films (plasticized with (**A**)—CCU and (**B**)—BU).

Figure 3 shows the thermal stability of the starch-based materials and composites. On the TGA curves representing the weight loss of the samples, a three-step degradation process is noticed. Additionally, some drop in the weight loss for the native starch step is attributed to moisture evaporation from the materials. For corn starch containing 10.9% moisture evaporated rapidly during the heating. However, moisture in the TPS films evaporated gradually because water molecules were bonded with hygroscopic compounds from the plasticizers. In the second step, there is barely visible with a peak with low intensity on DTG curves at ca. 200–209 °C that can be assigned to some decomposition of the plasticizer [10]. Third, the one with the highest intensity is related to decarbonization of the polysaccharide. Compared to the unmodified starch, TPS exhibited lower thermal stability. It is caused by a more amorphous structure of the starch [8,9,10,26] and the presence of a plasticizing mixture that is rich in U [10]. There is no significant difference in thermal stability comparing two types of the plasticizing mixtures (Supplementary Data—SD, Figure S1). However, the decarbonization of TPS with BU runs more gradually and slower (wider DTG peak). The presence of coffee filler did not affect the initial temperature of decomposition but there is some difference in T_deg0_ for two methods of the biocomposite preparation (Figure 3, SD Table S1). TPS/DES+cf exhibited a slightly higher thermal stability than TPS/DES/cf.

### 2.4. FTIR Characterization

**Figure 4:**
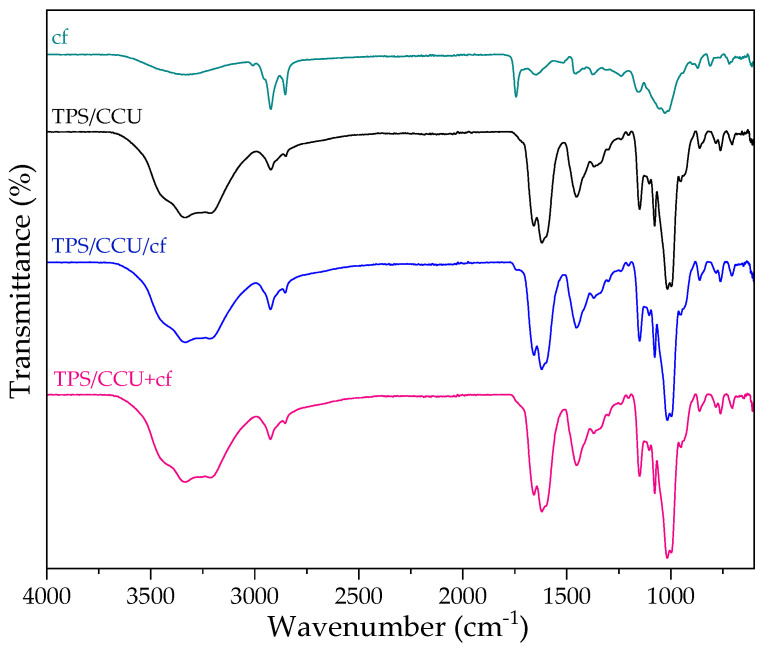
FTIR−ATR spectra of dried spent coffee grounds (cf), TPS/CCU and TPS/CCU with the filler.

Figure 4 shows FTIR-ATR spectra for dried spent coffee grounds, TPS CCU and two types of biocomposites. On the FTIR spectrum of cf, there is a broad band observed at 3680–2993 cm^−^^1^, which is assigned to the stretching of the O-H group due to the inter-molecular, but mainly, intra-molecular hydrogen bonding of polysaccharides, e.g., cellulose [35]. The band at 2926 cm^−^^1^ indicates the presence of a symmetric or asymmetric stretching vibration of C-H in aliphatic chains of coffee oils. The absorption peaks at 1748 cm^−^^1^ were assigned to the carboxyl linkage of xanthine derivatives (e.g., chlorogenic acids and caffeine) [36]. The sharp band between 1194 and 925 cm^−1^ is assigned to a stretching vibration of C-O in C-O-H bonds of glycosidic bonds. The spectra of TPS/CCU is described in previous work [10]. FTIR spectra confirm a presence of CCU in starch matrix. Comparing TPS/DES with composites, there are no new peaks indicating a lack of formation of new bonds and starch derivatization. There are also no shifts in the fingerprint region for starch (range: 1200–900 cm^−1^) indicating a strong H-bond formation between polysaccharide and additives [8]. It can be caused by a high DES content (60 pph) that can partially hinder to form bonding between cf and starch. It can be related to the retention of biocomposites’ flexibility (Figure 1).

### 2.5. Moisture Sorption, Swelling and Dissolution Degrees

Table 1 shows the results related to the behavior of samples in moisture and water. Due to the high urea content and highly amorphous structure of the polysaccharide caused by shearing forces from the extrusion process in the presence of DES [10], the swelling (240–375%) and dissolution (43–48%) degrees of the TPS/DES are much higher than the starch plasticized with conventional plasticizers based on polyols [37]. The filler addition into starch-based materials slightly increased the moisture’s sorption that can be caused by the hydrophilic character of spent coffee grounds and a high amount of quite hygroscopic plasticizer. However, the composites exhibited a lower swelling degree than TPS/DES films due to the presence of the solid filler as well as a lower amount of plasticizer in the final material.

### 2.6. Biodegradation in Soil

**Figure 5:**
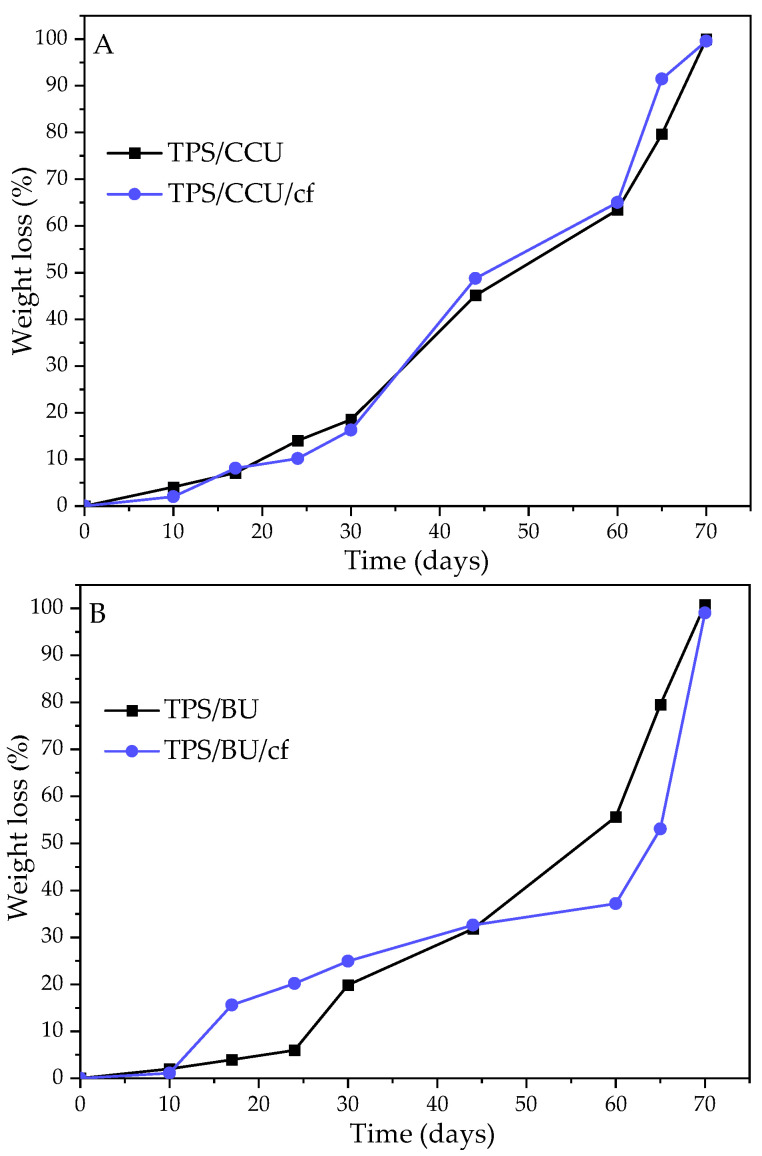
Degrees of biodegradation in soil of films and their biocomposites plasticized with (**A**)—CCU and (**B**)—BU.

The biodegradation test results for the TPS films in soil are presented in Figure 5. It can be noticed that all the investigated samples wholly degraded within 70 days, so it means that they degraded almost 3 weeks faster than the standard for the biodegradable materials requires. There is no significant difference between the TPS with two types of plasticizers. However, samples with BU degraded slightly slower. It can be related to a lower swelling degree for TPS/BUs than for TPS/CCUs (Table 1). Filler addition did not affect the biodegradation degree (the run of the changes in the process are different for samples with BU, but total disappearance is the same for all tested materials).

### 2.7. Influence of TPS Materials Presence on Physicochemical State of Model Plants

From data collected in Table 2, it can be noticed that the transpiration intensity for TPS/BU/cf as well as assimilation intensity for TPS/BU increased in the studied plants. Additionally, all materials except for TPS/CCU led to a decrease in substomatal CO_2_ concentration. Comparing the gas exchange parameters, the plants cultivated in the enriched substrate with the TPS/CCU presence did not vary from the plants grown in the control soil.

Comparing the influence of all samples from the enriched substrate with the control on the values of chlorophyll “a” fluorescence, an impact was observed only for TPS/BU; plants cultivated on the substrate enriched with this film exhibited higher initial fluorescence (F_0_) than the control (Table 3). High F_0_ values might indicate a decrease in the efficiency of the photosynthetic apparatus in the transfer of excitation energy between chlorophyll molecules. However, F_V_/F_M_ is considered the most reliable indicator of photosynthetic camera activity [38]. Angelini and coworkers [39] suggest that the F_V_/F_M_ index in plants in full development, under stress-free conditions, exhibits values of about 0.83, and according to Bjorkman and Demmig (1987)—0.78 to 0.84— this value clearly depends on genetic conditions [40]. In the case of plants growing on enriched soil with TPS/BU, it deviates from this range; however, it does not differ significantly from other experimental variants, which suggests that this additive has no toxic effect on the functioning of the photosynthetic apparatus of bean plants.

A significant effect of the applied film additives on the substrate on the content of assimilation pigments in the bean leaves was demonstrated (Table 4). Plants cultivated with the addition of TPS/CCU showed a reduced content of chlorophyll “b” compared to the control, which may suggest a decrease in the photosynthetic activity of these plants. In addition, higher levels of chlorophyll “a” and “b” and total chlorophyll have been shown in plants grown on substrate enriched with biocomposite films (TPS with cf) suggesting a greater effectiveness of the addition of these materials. Proline, on the other hand, is an amino acid that increases in content in plant tissue indicating the occurrence of some stress conditions [41]. The absence of significant differences between the tested variants in the case of proline content in bean leaves therefore suggests the absence of stress in plants.

The additives used for the soil substrate affected the growth and development of the plants. Potential slight toxic effects of TPS/BU on the growth and development of bean plants have been demonstrated. TPS/CCU/cf seems to be the best fertilizing additive due to the high values of plant physiology parameters. Nonetheless, further research is necessary to confirm this hypothesis.

### 2.8. Remaining of Nitrogen in the Soil Substrate from TPS Materials

**Figure 6:**
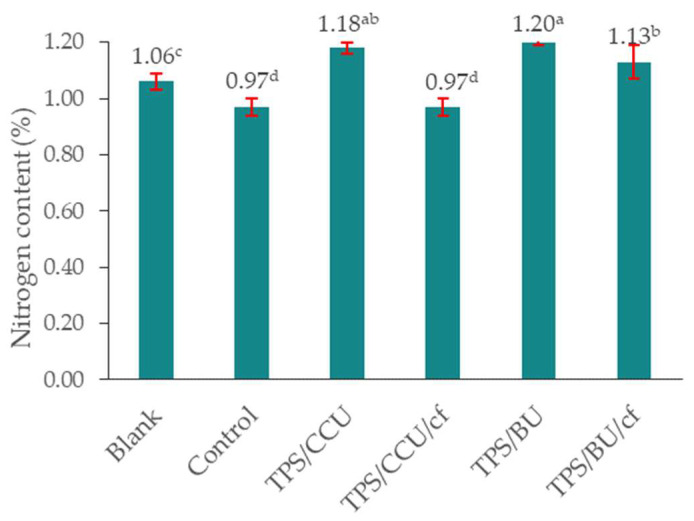
Total content of nitrogen in soil after model plant cultivation. “Blank” is a sample of pure, native soil (a–d—averages marked with the same letters do not differ significantly from each other for *p* < 0.05).

The percentage of total nitrogen content in the enriched soil substrate (collected after plant growth) in comparison with the control increased, which indicates the effective release of nitrogen from the TPS materials into the substrate (Figure 6). The highest nitrogen content was found in the enriched sample where films without filler were added (TPS/CCU 1.18% and TPS BU 1.20%). It can be related to a higher plasticizer content in the TPS films (without addition of 20 pph of filler) leading to an increase in U-rich plasticizer amount in the final biocomposite. No significant changes in the nitrogen content in the substrate enriched with TPS/CCU/cf were found. Comparing the type of additives, the higher nitrogen release was obtained for the TPS plasticized with the mixture of betaine and urea, than the system based on choline and urea. These preliminary tests highlight the necessity of a continuation of studies on the new TPS/DES materials as fertilizer carriers and their influence on different growing plants.

## 3. Materials and Methods

### 3.1. Materials

Common corn starch with a moisture content of 11 wt% (29.7 wt% amylose content) was supplied by Nowamyl SA (Nowogard, Poland). Urea—U (≥98%) was purchased from Chempur (Piekary Śląskie, Poland), anhydrous betaine—B (99%) from Alfa Aesar (Kandel, Germany) and choline chloride—CC (p.a.) from Aviresco (Solan, Ohio, USA). Spent coffee grounds were collected by the unit workers (finely ground coffee, 100% Arabica, Tschibo). For the studies on the toxicity and physiological state of growing plants, a yellow dwarf bean “Złota Saxa” (*Phaseolus vulgaris* L. cv. Złota Saxa) obtained from the seeds of the Verve company (Cracow, Poland) was used as the model plant. 

### 3.2. TPS Films Preparation

TPS composite films were prepared via two methods depending on two ways for the filler introduction. The first ones were prepared as follows: plasticizing systems: CC and U or B mixed at 1:5 molar ratio (60 pph of the mixture per dry starch), starch and dried spent coffee grounds (20 pph per dry starch) were mixed and kept in sealed polyethylene (PE) bags for 1 day before extrusion. These samples are named TPS/CCU/cf and TPS/BU/cf. In the second method, the filler was mixed with DES formed with CC and U or B (the components at 1:5 molar ratio and heated together until liquid was obtained) and heated at 80 °C for 2 h, then the whole system was added into starch and stored in sealed PE bags for 1 day in ambient conditions before further processing. The samples prepared according to this method were named TPS/CCU+cf and TPS/BU+cf. The premixtures without the filler were stored before extrusion in the same conditions as for the premixtures for composites.

**Scheme 2:**
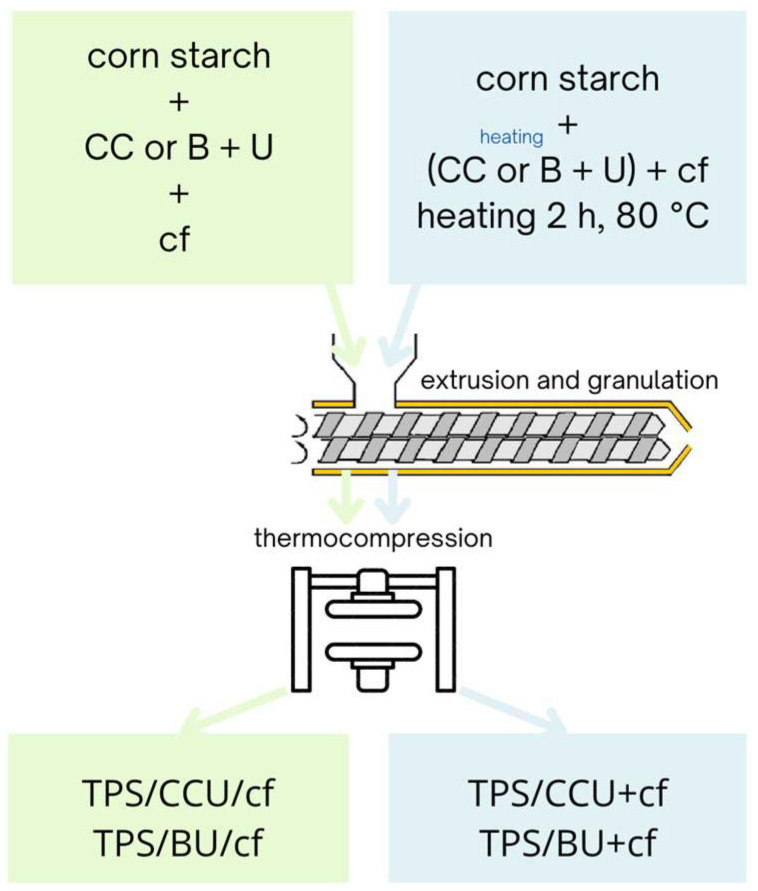
Scheme of preparation of the biocomposites.

Subsequently, all premixtures were thermally processed with a twin-screw co-rotational extruder with L/D 40:1 (LabTech Engineering, Samut Prakan, Thailand). The temperature profile of the extrusion was as follows: 60/90/100/105/105/105/105/105/105/105 °C, and the screw’s rotational speed was 90 rpm. Then, extrudates were stored in PE bags for a few days, granulated and thermocompressed to samples with dimensions 200 × 200 mm using hydraulic press (Remi-Plast, Czerwonak, Poland) at 125 °C, 153 bar for 10 s and cooled until reaching 80 °C under the pressure. The thickness of the films was 0.61–0.65 mm ±0.035. Before testing, samples were kept in a climate room at 23 °C, 50% RH for 1 week at least. Scheme 2 depicts the preparation of the biocomposites. 

### 3.3. Mechanical Tests

Mechanical tests for TPS films were performed using Zwick//Roell Z2.5 machine (load cell 2.5 kN, ZwickRoell GmbH & Co. KG, Ulm, Germany) according to standard ASTM D822-02. The films (thickness 0.65–0.73 mm) were cut into 10 mm wide strips. The initial grip separation was 50 mm, and the crosshead speed was 100 mm/min. At least 10 replicated samples for each system were tested, and the mechanical parameters (EB—elongation at break, TS—tensile strength and YM—Young’s modulus) were calculated with the TestXpert II software. 

### 3.4. Dynamical Mechanical Thermal Analysis—DMTA

The tests were conducted using a Dynamic Mechanical Analyzer Q800 (TA Instruments, New Castle, DE, USA). The value of the storage modulus (E′), loss modulus (E″) and the tangent of the loss angle (tanδ) depending on the temperature were determined. The test was made with a tension mode (measuring length 7–8 mm, specimen width 10 mm, thickness 0.60–0.65 mm), with a strain of 5 μm in the temperature range of −80–140 °C with a heating rate of 3 °C/min. The strain was set with a frequency of 1 Hz. 

### 3.5. Thermal Gravimetry Analysis—TGA

The thermal stability of native corn starch, TPS/DES and biocomposite films was investigated using TGA Q500 (TA Instruments, New Castle, DE, USA). Tests of ca. 10 mg of the samples were performed on platinum pans under 25 mL/min airflow, in the temperature range of 30–700 °C at a heating rate of 10 °C/min in an air atmosphere.

### 3.6. FTIR-ATR Spectroscopy of the Films

FTIR analysis of the filler and the films obtained via test 1 method was performed using the FT-IR spectrophotometer (Perkin Elmer, Spectrum 100, Waltham, MA, USA) equipped with ATR. For each sample, 12 scans were obtained in the range 4000–550 cm^−^^1^. Spectra were analyzed using the OMNIC software.

### 3.7. Evaluation of the Samples’ Behavior in Moisture and Water

For moisture absorption (A_t_) tests of the obtained materials, three samples (25 × 25 mm) were cut and dried for about 2 h (100 °C) to constant mass. The samples were then weighed and placed in a climate chamber (relative humidity—RH 50 ± 2%, temperature 25 ± 2 °C). The samples were weighed after 24 h of storage in the climatic chamber. The results obtained in this way were substituted for the formula [42]:

A_t_ = [(m_t_ − m_0_)/m_0_] × 100% (1)

where A_t_—sorption of moisture after time t [%]; m_0_—mass of the dry sample [g]; m_t_—sample mass after 24 h [g].

The solubility (Sol) in water of the materials was performed as followed: three samples (25 × 25 mm) were cut from the film and placed in a desiccator to remove moisture (to constant mass). The samples were then weighed, placed in plastic vials and filled with 50 mL of distilled water. After 24 h, the samples were removed and dried for about 2 h (100 °C) to a constant mass. The dry samples were weighed again. The solubility in water values were calculated using the following formula [42,43]:

Sol = [(m_1_ − m_2_)/m_1_] × 100% (2)

where Sol—solubility in water [%]; m_1_—mass of the dry sample [g]; m_2_—mass of the sample after drying [g].

The swelling degree (Sw) was measured as a change in mass (mass swelling). Three samples (25 × 25 mm) were cut from the film and placed in a drier to remove moisture (to constant mass). The samples were then weighed, placed in plastic vials and filled with 50 mL of distilled water. After 24 h, the samples were removed and dried on lint-free paper. Then, depending on the type of swelling, the mass of the sample or its surface was measured. The swelling in water values was calculated using the following formula:

Sw = [(m_1_ − m_2_)/m_1_] × 100% (3)

where Sw (total swelling matter)—swelling in water [%]; m_1_—mass of the dry sample [g] or surface of the dry sample [cm^2^]; m_2_—mass of the sample after drying [g] or mass of the sample after drying [cm^2^].

### 3.8. Determination of Biodegradability in Soil

The biodegradability and compostability of starch-based materials were evaluated under controlled composting conditions based on standard PN-EN 14046 “Evaluation of the ultimate aerobic biodegradability of packaging materials under controlled composting conditions. Method by analysis of released carbon dioxide”. The tested samples were cut into pieces with a size of approx. 15 × 15 mm. The samples were mixed in a ratio of 6:1 with compost and incubated at a constant temperature of 25 ± 2 °C. The vessel was refilled with ¾ of a mixture to allow aeration by hand shaking. The humidity of the compost was approx. 55% and pH = 7.3. In the following part of the study, the loss of weight was verified. 

### 3.9. Investigation of the Toxicity and Influence of the TPS-Based Films on Growing Plants’ Physiological State

At the beginning of June, the seeds (3 pots per variant, 2 seeds per pot) were sown in soil intended for the sowing and quilting of the “ATHENA” brand mixed in a ratio of 1:4 with perlite.

Individual sheets of films were cut into 10 × 10 mm fragments and mixed together with the prepared substrate before sowing. During the experiment, the water potential was maintained at −10 kPa in control conditions (optimal soil moisture) and −30 kPa in conditions of water deficit. The need for irrigation of plants was determined on the basis of contact soil tensiometers placed in pots of individual experimental variants at a depth of 20 cm. Irrigation was carried out using a drip line. 

#### 3.9.1. Gas Exchange Parameters

Measurements of the gas exchange parameters, i.e., assimilation intensity CO_2_ net (P_n_), transpiration intensity (E), stomatal conductivity H_2_O (g_s_) and substomatal CO_2_ concentration (c_i_), were made using a TPS-2 portable gas analyzer with a PLC-4 (PP Systems, Amesbury, MA, USA). The measurement was carried out in each variant on 15 randomly selected, fully grown dwarf bean leaves (the repetition was the measurement on one leaf). The photosynthetic water utilization factor (WUE) was determined on the basis of the P_n_/E quotient.

#### 3.9.2. Chlorophyll “a” Fluorescence Parameters

The fluorescence parameters of chlorophyll “a” were determined using a Handy PEA spectrofluorometer (Hansatech Ltd., Kings Lynn, UK), based on the standard procedure of the apparatus (3 × 650 nm LED, maximum actinic light intensity 3000 μmol m^−2^ s^−1^). The measurement was carried out in each variant on 15 randomly selected, fully grown bean leaves (the repetition was the measurement on one leaf), in a place previously darkened for 20 min, using factory-made clips (the irradiation area was 4 mm). The following parameters were measured: initial (zero) fluorescence, index of excitation energy loss in power antennas (F_0_), maximum fluorescence, after reduction of acceptors in PS II and after darkroom adaptation (F_M_), variable fluorescence, determined after dark adaptation (the parameter depends on maximum quantum efficiency of PS II) (F_V_ = F_M_ − F_0_), maximum potential efficiency of the photochemical reaction in PS II, determined after dark adaptation, after reduction of acceptors in PS II (F_V_/F_M_), chlorophyll fluorescence growth time from the beginning of the measurement to reaching maximum (T_FM_) and the area above the fluorescence induction curve of chlorophyll “a” between points F_0_ and F_M_ proportional to the size of the pool of reduced plastoquinone electron acceptors in PSII (A_M_ (Area)).

#### 3.9.3. Content of Photosynthetic Pigments

Determination of the content of photosynthetic pigments was performed by the method of Arnon et al. (1956) [44] modified by Lichtenthaler and Wellburn (1983) [45] for chlorophyll “a”, “b” and total chlorophyll, and the method of Hager and Mayer-Berthenrath (1966) [46] for carotenoids. The material (15 fully developed, randomly selected leaves) was collected from each experimental variant, which were then divided into smaller fragments. From the prepared material, 0.03 g of fresh mass was obtained for the extraction of pigments. The samples were ground in a mortar with 10 cm^3^ of 80% acetone. The solutions were placed in test tubes and then transferred to a centrifuge to separate the liquid phase from the solid phase. Centrifugation was carried out for 10 min at 1500 rpm. The optical density of the samples was determined using a Shimadzu UV-1280 spectrophotometer (Japan). Determinations were made at wavelengths: 440, 645 and 663 nm. The content of dyes was calculated according to the formulas:

µg of chlorophyll “a” × g^−^^1^ FM [12.7 (Ek 663) − 2.69 (Ek 645)] × V/W, (4)

µg of total chlorophyll × g^−^^1^ FM [20.2 (Ek 645) + 8.02 (Ek 663)] × V/W, (5)

µg of carotenoids × g^−^^1^ FM [4.16 (Ek 440) − 0.89 (Ek 663)] × V/W, (6)

where Ek—extinction at a certain wavelength, V—amount of 80% acetone used for extraction and W—mass of fresh sample in grams, FM —fresh mass.

#### 3.9.4. Determination of Proline Content

The content of free proline in plant tissue was determined by the method of Bates et al. [47], consisting of measuring the absorbance of a colored complex of proline and acidic ninhydrin extracted with toluene, at the wavelength of λ = 520 nm. Plant tissue (0.5 g) was homogenized with 10 cm^3^ of 3% sulfosalicylic acid. The proline content was recalculated from the standard curve and expressed in mg·g^−1^ FM plants.

### 3.10. Nitrogen Content in Soil Substrate

Nitrogen content in soil was determined according to the standard PN-EN 16168:201 (‘Sludge, treated biowaste and soil—Determination of total nitrogen using dry combustion method”). For nitrogen determination, soil samples were ground in a porcelain mortar to grains with a diameter of less than 0.1 mm. Total content of nitrogen (N_tot_) was analyzed using the elemental CHNS/O analyzer (Flash*Smart* Thermo Scientific, Waltham, MA, USA, Costech Instruments Elemental Combustion System, Alexandria, VI, USA).

### 3.11. Statistical Analysis

The results of the study were statistically analyzed in STATISTICA version 13.3 (StatSoft, Poland) using one-way analysis of variance tests in a completely randomized design. The significance of the differences between the means was determined using the Duncan test, with the significance level of α = 0.05. The same one-letter markings were used to indicate means that did not differ statistically from each other.

## 4. Conclusions

The mixture of choline chloride or betaine with an excess of urea effectively plasticized corn starch and flexible, highly transparent films based on thermoplastic starch were obtained. However, the BU mixture exhibited some tendency to recrystallize in the polysaccharide matrix. The addition of a high amount of spent coffee grounds (20 pph per dry starch) as a filler into TPS/DES increased the mechanical properties but did not affect the processability of the materials as the DMTA results revealed. A better reinforcement effect was obtained for the starch plasticizer with BU than CCU. The sorption degrees of the biocomposites were decreased for the biocomposites. However, the parameters’ values were quite high, indicating a gel formation being caused with a highly amorphous structure of the materials and U presence. The studies on the influence of the TPS materials in soil on the physiological state revealed that our products, especially plasticized with CCU, did not exhibit a toxic effect on the yellow dwarf bean. The percentage of total nitrogen in the soil substrate in comparison with the control increased, indicating an effective release of nitrogen from the TPS materials into the substrate.

The obtained materials can be successfully applied as fertilizer carriers and agriculture bioplastics.

## Figures and Tables

**Table 1:** Moisture (A_t_) at RH 50%, swelling (Sw) and solubility degrees (Sol) in distilled water of the materials after 24 h of storage in RH or distilled water.

Sample	A_t RH50%_ (%)	Sw (%)	Sol (%)
TPS/CCU	13.2 (±0.001)	375 (±0.06)	46 (±0.01)
TPS/CCU/cf	14.4 (±0.000)	275 (±0.22)	48 (±0.01)
TPS/CCU+cf	14.7 (±0.000)	240 (±0.21)	45 (±0.05)
TPS/BU	14.2 (±0.004)	349 (±0.53)	48 (±0.01)
TPS/BU/cf	15.8 (±0.000)	283 (±0.07)	46 (±0.02)
TPS/BU+cf	15.5 (±0.004)	277 (±0.02)	43 (±0.02)

**Table 2:** Gas exchange parameters for leaves of *Phaseolus vulgaris* L. cultivated on soil substrate enriched with TPS-based films.

Sample	Transpiration Intensity (mmol H_2_O∙m^−2^∙s^−1^)	Stomal Conductivity H_2_O (mol H_2_O∙m^−2^∙s^−1^)	Assimilation Intensity CO_2_ Net (mmol H_2_O∙m^−2^∙s^−1^)	Substomatal CO_2_ Concentration (μmol CO_2_∙mol^−1^)
Control	0.393 (±0.093) ^b^	0.036 (±0.015) ^ab^	2.508 (±0.775) ^bc^	414.67 (±37.31) ^a^
TPS/CCU	0.489 (±0.121) ^ab^	0.042 (±0.013) ^a^	2.633 (±0.735) ^bc^	312.83 (±53.90) ^b^
TPS/CCU/cf	0.348 (±0.092) ^b^	0.025 (±0.006) ^b^	2.217 (±0.904) ^c^	372.58 (±77.34) ^a^
TPS/BU	0.405 (±0.087) ^a^	0.032 (±0.010) ^ab^	2.792 (±1.133) ^a^	284.17 (±72.82) ^c^
TPS/BU/cf	0.372 (±0.092) ^a^	0.031 (±0.009) ^a^	3.000 (±0.283) ^bc^	269.09 (±41.98) ^b^

a–c—averages marked with the same letters do not differ significantly from each other for *p* < 0.05

**Table 3:** Fluorescence parameters of chlorophyll “a” in leaves of *Phaseolus vulgaris* L. growing on the substrate enriched with TPS-based films.

Sample	Initial (zero) Fluorescence F_0_	Maximum Fluorescence F_M_	Variable Fluorescence F_V_	F_V_/F_M_	Chlorophyll Fluorescence Growth Time (ms)	Area above the Fluorescence Induction Curve (kbms)
Control	239.50 (±18.75) ^b^	1119.08 (±120.12) ^a^	895.25 (±99.22) ^a^	0.782 (±0.046) ^a^	866.67 (±49.24) ^a^	52.89 (±73.81) ^ab^
TPS/CCU	224.46 (±9.90) ^b^	1190.54 (±53.16) ^ab^	966.08 (±53.33) ^a^	0.811(±0.011) ^a^	900.00 (±0.00) ^a^	64.20 (±55.18) ^a^
TPS/CCU/cf	226.09 (±11.00) ^b^	1119.00 (±103.09) ^a^	917.27 (±124.69) ^a^	0.800 (±0.022) ^a^	872.73 (±64.67) ^a^	51.97 (±77.45) ^a^
TPS/BU	268.88 (±53.46) ^a^	1084.38 (±242.47) ^a^	815.50 (±278.63) ^a^	0.733 (±0.109) ^a^	862.50 (±51.75) ^a^	40.51 (±18.51) ^b^
TPS/BU/cf	232.17 (±19.06) ^b^	1179.08 (±102.33) ^a^	946.92 (±110.28) ^a^	0.801 (±0.027) ^a^	883.33 (±38.92) ^a^	59.83 (±10.73) ^a^

a–b—averages marked with the same letters do not differ significantly from each other for *p* < 0.05

**Table 4:** Content of assimilation pigments and proline in leaves of *Phaseolus vulgaris* L. growing on a substrate enriched with TPS-based films.

Sample	Chlorophyll “a” (mg g^−1^ FM)	Chlorophyll “b” (mg g^−1^ FM)	Total Chlorophyll (mg g^−1^ FM)	Carotenoids (mg g^−1^ FM)	Proline (mg g^−1^ FM)
Control	2.88 (±0.19) ^abc^	1.37 (±0.20) ^ab^	4.25 (±0.22) ^abc^	2.20 (±0.02) ^a^	0.58 (±0.05) ^a^
TPS/CCU	2.60 (±0.73) ^ac^	1.09 (±0.30) ^c^	3.69 (±1.01) ^c^	1.76 (±0.72) ^a^	0.64 (±0.04) ^a^
TPS/CCU/cf	3.45 (±0.16) ^ab^	1.41 (±0.05) ^b^	4.86 (±0.18) ^ab^	2.56 (±0.29) ^a^	0.62 (±0.03) ^a^
TPS/BU	2.63 (±0.40) ^bc^	1.12 (±0.23) ^bc^	3.75 (±0.62) ^bc^	1.62(±0.39) ^a^	0.63 (±0.04) ^a^
TPS/BU/cf	3.50 (±0.17) ^a^	1.51 (±0.12) ^a^	5.01 (±0.19) ^a^	2.76 (±0.74) ^a^	0.65 (±0.07) ^a^

a–c—averages marked with the same letters do not differ significantly from each other for *p* < 0.05.

## Data Availability

Not applicable.

## Supplementary Materials

The following supporting information (TGA curves) can be downloaded at: https://www.mdpi.com/article/10.3390/ijms24097864/s1.
