# Supplementary material for: Study on Physicochemical Properties of Biocomposite Films with Spent Coffee Grounds as a Filler and Their Influence on Physiological State of Growing Plants
Source: Int J Mol Sci. 2023 Apr 26;24(9):7864. doi: 10.3390/ijms24097864 (PMC10178467; doi:10.3390/ijms24097864)
Supplement: Supplementary file 1 [file ijms-24-07864-s001.zip › ijms-2347049-supplementary.pdf]

Supplementary Data

Study on Physicochemical Properties of Biocomposite Films with Spent Coffee Grounds as a Filler and their Influence on Physiological State of Growing Plants

Magdalena Zdanowicz [1,*], Marta Rokosa [2], Magdalena Pieczykolan [1], Adrian Krzysztof Antosik [1,3], Justyna Chudecka [4] and Małgorzata Mikiciuk [2]

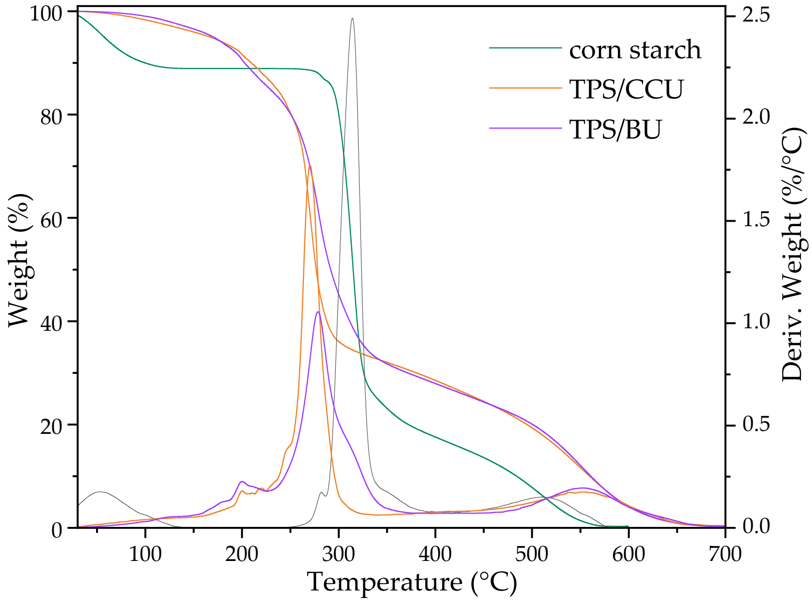

Figure S1. Thermal results (TGA and DTG curves) for native corn starch and TPS/DES.

Table S1. Temperature of drop of the weight loss – initial temperature of a decomposition (T_{deg0}) and temperature for maximum peak on DTG.

Sample	T_{deg0} (° C)	DTG $_{max\ peak}$ (° C)
Starch	292	314
TPS/CCU	239	272
TPS/CCU/cf	251	272
TPS/CCU+cf	244	274
TPS/BU	247	278
TPS/BU/cf	245	275
TPS/BU+cf	243	280
